# AAV-PHP.eB transduces both the inner and outer retina with high efficacy in mice

**DOI:** 10.1016/j.omtm.2022.03.016

**Published:** 2022-03-28

**Authors:** Arpad Palfi, Naomi Chadderton, Sophia Millington-Ward, Iris Post, Pete Humphries, Paul F. Kenna, G. Jane Farrar

**Affiliations:** 1 Department of Genetics, School of Genetics and Microbiology, Trinity College Dublin, D02 VF25, Dublin, Ireland; 2 The Research Foundation, Royal Victoria Eye and Ear Hospital, D02 XK51, Dublin, Ireland

**Keywords:** retina, eye, mouse, AAV2/2, AAV2/8, AAV-PHP.eB, retinal degeneration, gene therapy, EGFP

## Abstract

Recombinant adeno-associated virus (AAV) vectors are one of the main gene delivery vehicles used in retinal gene therapy approaches; however, there is a need to further improve the efficacy, tropism, and safety of these vectors. In this study, using a CMV-EGFP expression cassette, we characterize the retinal utility of AAV-PHP.eB, a serotype recently developed by *in vivo* directed evolution, which can cross the blood-brain barrier and target neurons with high efficacy in mice. Systemic and intravitreal delivery of AAV-PHP.eB resulted in the high transduction efficacy of retinal ganglion and horizontal cells, with systemic delivery providing pan-retinal coverage of the mouse retina. Subretinal delivery transduced photoreceptors and retinal pigment epithelium cells robustly. EGFP expression (number of transduced cells and mRNA levels) were similar when the retinas were transduced systemically or intravitreally with AAV-PHP.eB or intravitreally with AAV2/2. Notably, in photoreceptors, EGFP fluorescence intensities and mRNA levels were 50–70 times higher, when subretinal injections with AAV-PHP.eB were compared to AAV2/8. Our results demonstrate the pan-retinal transduction of ganglion cells and extremely efficient transduction of photoreceptor and retinal pigment epithelium cells as the most valuable features of AAV-PHP.eB in the mouse retina.

## Graphical abstract

**Figure:**
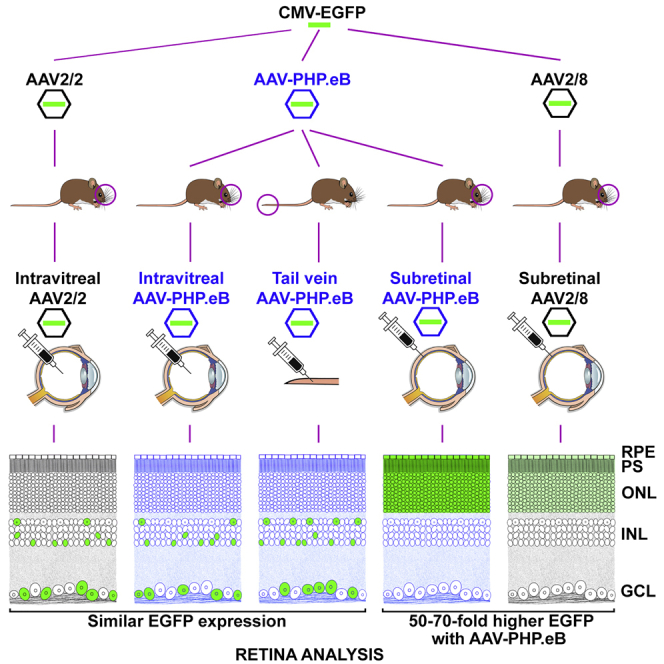

## Teaser

AAV-PHP.eB transduces the brain efficiently in mice. Palfi et al. provide a detailed transduction profile for AAV-PHP.eB in mouse retina following systemic and intraocular delivery. Subretinal delivery with AAV-PHP.eB transduced photoreceptors 50- to 70-fold greater than the benchmark serotype, AAV2/8. Such efficacy should reduce AAV dose requirements, increasing the safety of gene delivery.

## Introduction

With more than 30 completed or ongoing clinical trials (ClinicalTrials.gov) and many more in the pipeline, AAV-delivered retinal gene therapies promise treatments for visual impairments that are untreatable with existing conventional therapeutics. Data regarding safety and efficacy of AAV-gene therapies have accrued rapidly and can be exemplified with the success of Luxturna, the first authorized retinal gene therapy targeting RPE (retinal pigment epithelium)65-linked retinal dystrophy.^1^^,^^2^ Mutations in more than 300 genes are causative of inherited retinal degenerations (IRDs).^3^, ^4^, ^5^ Mutations in the more common IRD genes often lead to photoreceptor degeneration.^5^, ^6^, ^7^, ^8^, ^9^ In rodent models, non-human primates (NHPs), and humans, delivering gene therapies to photoreceptors is highly efficient via subretinal (SR) injection of various AAV serotypes, including AAV2, AAV5, AAV8, AAV9, and AAVrh10.^10^, ^11^, ^12^, ^13^ However, mutations in many other IRD genes are expressed and/or affect non-photoreceptor cells.^5^^,^^8^^,^^14^, ^15^, ^16^, ^17^ Therefore, the availability of vectors enabling safe and efficient gene delivery to these cell types is also of paramount importance. For example, AAV2/2 is highly efficient at transducing retinal ganglion cells (RGCs) via intravitreal (IVT) injection.^18^, ^19^, ^20^, ^21^, ^22^

Apart from improving the efficacy and specificity of the transduction of various cell types, the evolution of AAV technology is driven by many other factors, such as minimizing a host immune response, improving the safety and efficacy of AAV delivery in clinical settings, and optimizing AAV production.^23^, ^24^, ^25^ In the retina, SR delivery is a technically and clinically challenging route of administration, especially when considered in the physical context of a retina with advanced degeneration in IRD patients.^25^, ^26^, ^27^ AAV delivery via an IVT route is less invasive, but it may provoke a more extensive immune response compared to SR delivery.^25^, ^26^, ^27^ While an increasing repertoire of AAV serotypes is available for SR and IVT delivery to the retina, there is still a significant focus on improving aspects of these vectors.^24^^,^^25^ For example, *in vivo* directed evolution has been used to engineer AAV variants, which exhibit enhanced retinal transduction via IVT delivery.^28^^,^^29^

In addition, the systemic delivery of AAV could provide non-invasive access to the retina at least in experimental model systems, yet may lead to the cotransduction of multiple organs. In this regard, AAV9 has been shown to target the central nervous system (CNS) efficiently via the systemic route.^28^^,^^30^, ^31^, ^32^, ^33^, ^34^ AAV-PHP.eB, a serotype recently derived by *in vivo* directed evolution of AAV9 and AAV-PHP.B, exhibits further enhanced tropism toward the CNS.^23^ Notably, initial results with tdTomato gene delivery suggests that AAV-PHP.eB also transduces the mouse retina efficiently.^35^ In particular, AAV-PHP.eB transduced photoreceptor cells and horizontal cells robustly via SR and systemic deliveries, respectively.^35^ A limited number of RGC (IVT delivery) and some cells in the bipolar and ganglion cell layers (systemic delivery) were also transduced, suggesting that AAV-PHP.eB can target various retinal cell types, albeit depending on the route of administration.^35^ As the original work with AAV-PHP.eB in the mouse retina was a proof-of-concept study with limited data presented, we decided to further explore the utility of this serotype in retinal gene delivery.^35^ Utilizing the AAV-PHP.eB capsid, we delivered a cytomegalovirus (CMV) promoter-driven EGFP expression cassette (CMV-EGFP) via systemic, IVT, and SR routes to the murine retina. We compared the tropism and efficacy of AAV-PHP.eB to IVT and SR deliveries of AAV2/2^36^, ^37^, ^38^ and AAV2/8^10^^,^^39^ capsids, respectively, which are highly efficient at transducing the retina via these routes.

## Results

In this study, we assessed the utility of the recently derived AAV-PHP.eB serotype for gene delivery to the retina.^23^ We analyzed EGFP expression in the murine retina transduced with AAV-PHP.eB-CMV-EGFP using different delivery routes, including systemic delivery via tail vein (TV) injection, as well as IVT and SR intraocular injections. Control AAVs with serotypes frequently used for intraocular administration, that is, AAV2/2-CMV-EGFP for IVT injection, and AAV2/8-CMV-EGFP for SR injection were used to enable the comparative analysis of the different serotypes.

### Systemic and intravitreal delivery

**Figure 1:**
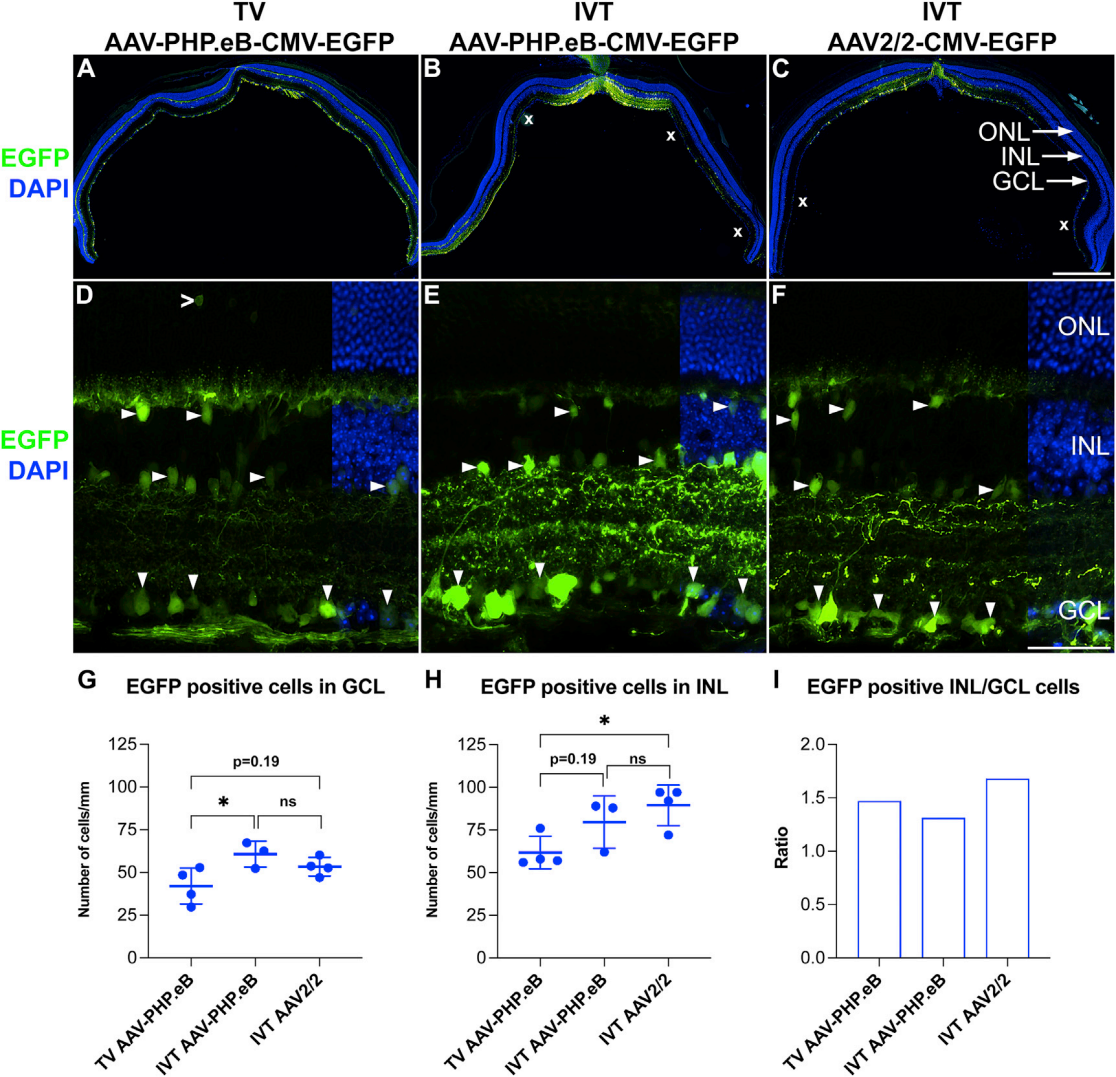
AAV-PHP.eB transduces the inner retina via systemic and intravitreal delivery Adult mice received 5.0 × 10^10^ vg/animal of AAV-PHP.eB-CMV-EGFP via tail vein (TV) injection or 7.5 × 10^8^ vg/eye of AAV-PHP.eB-CMV-EGFP or AAV2/2-CMV-EGFP (control) intravitreally (IVT; n = 3–4). Eyes were enucleated, fixed in 4% paraformaldehyde (PFA), and cryosectioned at 1 month post-delivery. (A–C) Overview of whole retina sections and (D–F) higher magnification of transduced areas; EGFP fluorescence (green) and DAPI nuclear counterstain (blue). In the transduced areas, EGFP^+^ cells were quantified in the ganglion cell layer (GCL; G) and the inner nuclear layer (INL; H); the ratio of the labeled cells in the INL versus GCL is shown in (I). x, areas with lower transduction (B and C). Downward arrowheads indicate examples of EGFP^+^ cells in the GCL; horizontal arrowheads indicate examples of EGFP^+^ cells in the INL; an open arrowhead indicates an EGFP^+^ photoreceptor cell (D). ONL, outer nuclear layer. Scale bars represent 500 μm (C) and 50 μm (F). ∗p < 0.05, ANOVA.

Adult 129 S2/SvHsd mice were used in this study as the 129 strains were permissive to PHP.B transduction across the blood-brain barrier.^36^ Mice were administered with 5.0 × 10^10^ vg of AAV-PHP.eB-CMV-EGFP via TV injection (TV AAV-PHP.eB-CMV-EGFP) or 7.5 × 10^8^ vg/eye of AAV-PHP.eB-CMV-EGFP or AAV2/2-CMV-EGFP via IVT injection (IVT AAV-PHP.eB-CMV-EGFP and IVT AAV2/2-CMV-EGFP, respectively; n = 3–4). AAV2/2 was selected as a control serotype as it is the serotype most extensively used for IVT delivery to RGCs in rodents, NHPs, and humans.^18^^,^^37^, ^38^, ^39^, ^40^, ^41^ As AAV2/2 does not cross the blood-brain barrier, it was not administered via TV.^42^ The effective dose of AAV-PHP.eB-CMV-EGFP was based on previous studies,^23^^,^^35^^,^^43^^,^^44^ and as it reflected dosage to the whole body, it was significantly higher than the dose range used for direct IVT delivery to the eye.^18^ EGFP expression from transduced retinas was analyzed by histology at 1 month post-AAV delivery (Figure 1). With both AAVs and delivery routes, significant EGFP expression was found in the inner retina (Figure 1), while very few labeled cells were detected in the outer retina (for the latter, an example is given in Figure 1D). If present, outer retina labeling was mostly detected in areas with very high transduction rates with IVT delivery. Notably, TV AAV-PHP.eB-CMV-EGFP resulted in an even transduction of the whole murine retina (Figure 1A). In contrast, IVT AAV-PHP.eB-CMV-EGFP and IVT AAV2/2-CMV-EGFP resulted in partial and uneven transduction of the retina (Figures 1B and 1C). The observed significant transduction via IVT AAV-PHP.eB-CMV-EGFP (Figure 1B) was unexpected as minimal transduction was observed via IVT AAV-PHP.eB previously.^35^ EGFP fluorescence in cells in the ganglion cell layer (GCL) was typically less intense in TV AAV-PHP.eB-CMV-EGFP transduced retinas (Figures 1D–1F). In the transduced areas, the number of EGFP^+^ cells in the GCL were slightly higher in the eyes transduced via IVT (IVT AAV-PHP.eB-CMV-EGFP: 60.6 ± 6.2 cells/mm, p < 0.05, ANOVA; IVT AAV2/2-CMV-EGFP: 53.4 ± 5.4 cells/mm, p = 0.19, ANOVA; Figure 1G) versus TV (TV AAV-PHP.eB-CMV-EGFP: 42.1 ± 10.6 cells/mm; Figure 1G) delivery. Similarly, the number of EGFP positive cells in the inner nuclear layer (INL) were also higher in the eyes transduced via IVT (IVT AAV-PHP.eB-CMV-EGFP: 79.6 ± 15.1 cells/mm, p = 0.19, ANOVA; IVT AAV2/2-CMV-EGFP: 89.7 ± 11.8 cells/mm, p < 0.05, ANOVA; Figure 1H) versus TV (TV AAV-PHP.eB-CMV-EGFP: 62.0 ± 9.1 cells/mm; Figure 1H) delivery. The difference in both EGFP intensities in cells and the EGFP^+^ cell numbers between TV and IVT injections may, in part, be a function of the doses delivered. It is therefore reasonable to assume that a higher TV dose could have resulted in higher EGFP intensities and number of EGFP^+^ cells. Meanwhile, the similar number of transduced cells between IVT AAV-PHP.eB-CMV-EGFP and IVT AAV2/2-CMV-EGFP deliveries (using the same dose) suggest similar efficacies for these serotypes. The ratios of the EGFP^+^ cells in the INL versus GCL were also similar for the three delivery combinations (Figure 1I), suggesting a generally similar tropisms for these AAVs and delivery routes.

**Figure 2:**
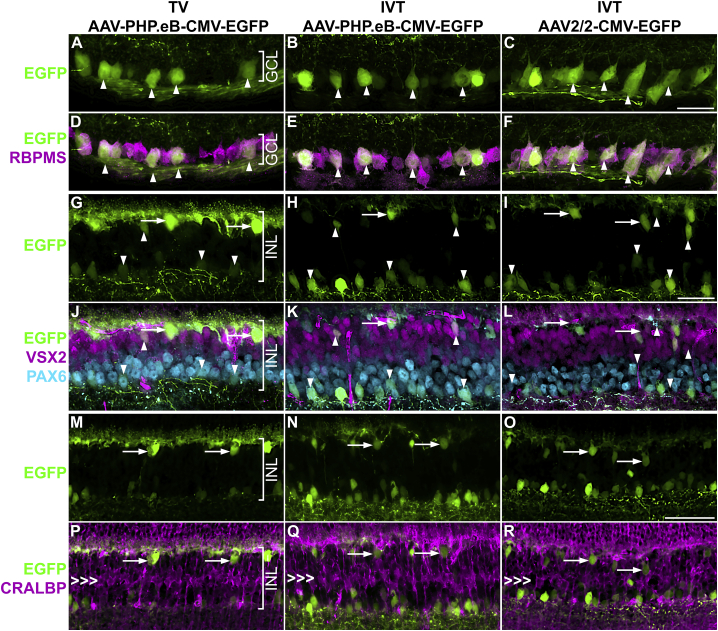
Colabeling of EGFP transduced cells with cell markers in the inner retina Adult mice received 5.0 × 10^10^ vg/animal of AAV-PHP.eB-CMV-EGFP via TV injection or 7.5 × 10^8^ vg/eye of AAV-PHP.eB-CMV-EGFP or AAV2/2-CMV-EGFP (control) IVT (n = 3–4). Eyes were enucleated, fixed in 4% PFA, cryosectioned, and stained with immunohistochemistry for retinal cell markers at 1 month post-delivery. (A–F) GCL. (A–C) EGFP fluorescence (green), (D–F) overlay of EGFP fluorescence (green), RBPMS immunohistochemistry (retinal ganglion cell marker, magenta), and nuclear counterstain (DAPI, blue). Arrowheads indicate examples of EGFP^+^ retinal ganglion cells. (G–R) INL. (G–I) EGFP fluorescence (green), (J–L) overlay of EGFP fluorescence (green), VSX2 immunohistochemistry (bipolar cell marker; magenta), and PAX6 immunohistochemistry (amacrine cell marker; light blue). Upward arrowheads indicate examples of EGFP^+^ bipolar cells; downward arrowheads indicate examples of EGFP^+^ amacrine cells, while arrows indicate examples of EGFP^+^ horizontal cells. (M–O) EGFP fluorescence (green). (P–R) Overlay of EGFP fluorescence (green) and CRALBP immunohistochemistry (Müller cell marker; magenta). Arrows indicate horizontal cells, while triple arrowheads mark the layer of Müller cell bodies. Scale bars represent 50 μm (C, I, and O).

To identify the retinal cell type of the EGFP^+^ cells, immunocytochemistry with various retinal cell markers, including RBPMS (RGC marker), PAX6 (RGC and amacrine cell marker), VSX2 (bipolar cell marker), and CRALBP (Müller cell marker) was carried out on the transduced retinal sections (Figure 2 and Table 1). For both routes of administration, TV and IVT, the RGCs (RBPMS^+^ cells) were efficiently transduced with AAV-PHP.eB-CMV-EGFP at the used doses (Figures 2A–2F); most of the labeled cells in the GCL were RGCs. RGC axons and dendrites were also labeled following both TV and IVT delivery (Figures 2A–2F; see also Figures 1D–1F). Colabelling revealed that some amacrine cells (PAX6^+^) and bipolar cells (VSX2^+^) were transduced in the INL in a similar fashion by TV AAV-PHP.eB-CMV-EGFP, IVT AAV-PHP.eB-CMV-EGFP, and IVT AAV2/2-CMV-EGFP (Figures 2G–2L). Horizontal cells (identified by their localization, shape, and arborization) were also targeted; the highest transduction level was found in retinas receiving TV AAV-PHP.eB-CMV-EGFP (Figures 2G–2I and 2M–2O). Müller cells (CRALBP^+^) were not transduced at all using either route of administration (Figures 2M–2R).

**Figure 3:**
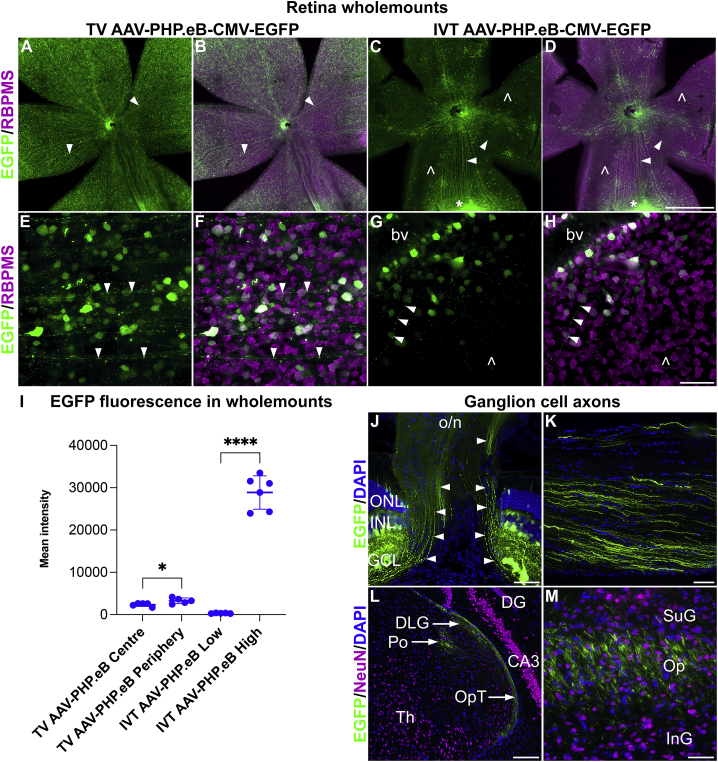
Transduction pattern in retinal ganglion cells following systemic and intravitreal delivery of AAV-PHP.eB 5.0 × 10^10^ vg/mouse and 7.5 × 10^8^ vg/eye of AAV-PHP.eB-CMV-EGFP was delivered to adult mice via the TV (n = 5) and IVT injection (n = 6), respectively. Eyes and brains were fixed in 4% PFA and cryosectioned at 1 month post-delivery; retinas were stained with EGFP (green) and RBPMS (magenta), and brains with NeuN (magenta) immunocytochemistry. (A–D) Overview of transduced retinal wholemounts. (E–H) Higher magnification of labeled cells. (A, C, E, and G) EGFP immunohistochemistry (green); (B, D, F, and H) overlay of EGFP (green) and RBPMS (ganglion cell marker; magenta) immunohistochemistry. Arrowheads: EGFP^+^ axons, empty arrowheads: EGFP^−^ areas. The asterisk indicates an area with very high EGFP expression (C and D). bv, blood vessel. Mean EGFP fluorescence intensities were quantified in TV and IVT injected eyes (I). For TV delivery, the central (TV AAV-PHP.eB Center; I) and peripheral (TV AAV-PHP.eB Periphery; I) areas were measured separately, while for IVT delivery, areas with low (IVT AAV-PHP.eB Low; I) and high intensities (IVT AAV-PHP.eB High; I) were quantified separately. EGFP fluorescence (green) in the transduced retinal ganglion cell axons (via IVT delivery of AAV-PHP.eB-CMV-EGFP) was tracked from the optic nerve head (J) via the optic nerve (K) and the optic tract (L) to the superior colliculus (M; n = 5). Neurons were labeled with NeuN immunohistochemistry (magenta) in the brain (L and M), and cell nuclei counterstained with DAPI (blue). Arrowheads indicate EGFP^+^ axons (J). CA3, field CA3 (hippocampus); DG, dentate gyrus (hippocampus); DLG, dorsal lateral geniculate nucleus (thalamus); GCL, ganglion cell layer (retina); InG, intermediate gray (superior colliculus); INL, inner nuclear layer (retina); o/n, optic nerve; ONL, outer nuclear layer (retina); Op, optic nerve layer (superior colliculus); OpT, optic tract; Po, posterior thalamic nuclei; SuG, superficial gray (superior colliculus); Th, thalamus. Scale bars represent 1 mm (D), 50 μm (H, J, and K), 100 μm (L), and 25 μm (M). ∗p < 0.05, ∗∗∗∗p < 0.0001, t test (I).

In another set of animals, the distribution of EGFP^+^ RGCs in the whole retina was compared using retina wholemounts transduced with 5.0× 10^10^ vg/mouse of TV AAV-PHP.eB-CMV-EGFP (n = 5) or 7.5 × 10^8^ vg/eye of IVT AAV-PHP.eB-CMV-EGFP (n = 6) at 1 month post-delivery. Retinas were stained with RBPMS (RGC marker) and EGFP immunohistochemistry to colocalize EGFP and RGCs. TV AAV-PHP.eB-CMV-EGFP transduced retinas displayed a more uniform and pan-retinal distribution of EGFP^+^ cells in the wholemounts (Figures 33A, 3B, 3E, and 3F), while IVT AAV-PHP.eB-CMV-EGFP transduced retinas (Figures 3C, 3D, 3G, and 3H) exhibited highly uneven EGFP expression, with some areas (including the optic nerve head region) expressing EGFP at high levels, while other areas displayed much lower EGFP fluorescence (Figures 3C, 3D, 3G, and 3H). Quantification of the mean EGFP fluorescence intensities in the transduced retinas is given in Figure 3I. In the TV-transduced retinas, the peripheral retina (3,275.8 ± 670.8, n = 5) exhibited slightly higher (+39.3%) fluorescence intensity levels compared to the central retina (2,351.2 ± 412.7, n = 5, p < 0.05, t test; Figure 3I). In the IVT-transduced retinas, the mean EGFP fluorescence intensity values were 28,869 ± 3,947.2 and 252.0 ± 178.6 for the high- and low-intensity areas, respectively, representing a 114.6-fold difference (n = 6, p < 0.0001, t test; Figure 3I).

Apart from the RGC cell bodies (Figures 3E–3H), RGC axons were also labeled with EGFP (Figures 3A–3H) using both TV and IVT routes of administration. The EGFP^+^ RGC axons could be identified extending from the retina via the optic nerve head (Figure 3J), optic nerve (Figure 3K), and optic tract (Figure 3L) reaching the thalamic nuclei (Figure 3L) and superior colliculi (Figure 3M). To track EGFP^+^ RGC axons in the brain, IVT AAV-PHP.eB-CMV-EGFP mice were analyzed as various cells, including many neurons in the brain, were transduced in mice given TV AAV-PHP.eB-CMV-EGFP (data not shown). If TV AAV-PHP.eB-CMV-EGFP mice had been used, then fluorescence from TV AAV-PHP.eB-CMV-EGFP transduced cells and their processes in the brain could have interfered with the identification of the EGFP label originating from the retina.

### Subretinal delivery

**Figure 4:**
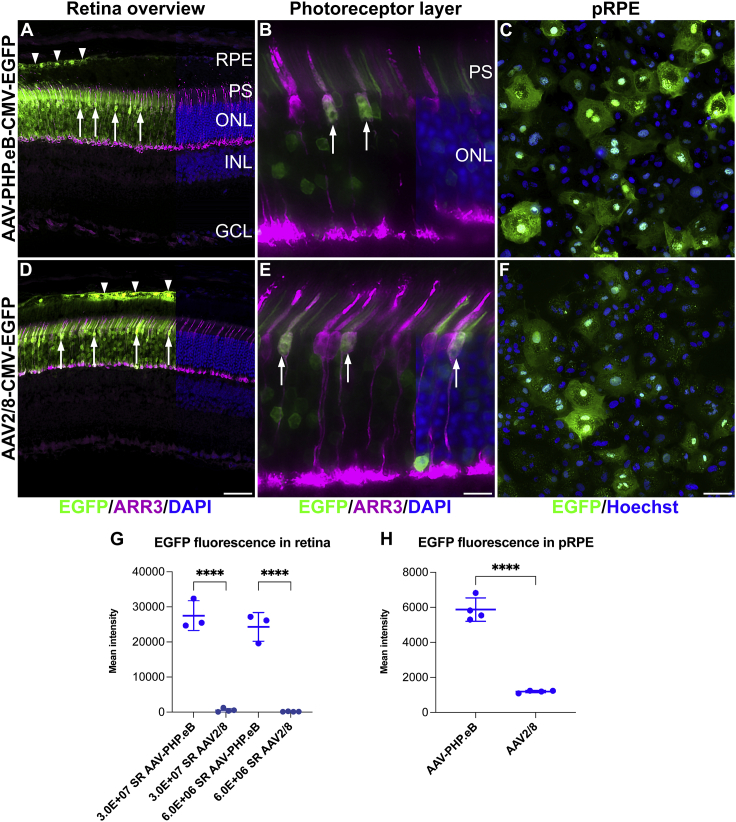
AAV-PHP.eB efficiently transduces rod and cone photoreceptors and retinal pigment epithelium cells Adult mouse eyes were injected subretinally with 3.0 × 10^7^ and 6.0 × 10^6^ vg/eye of AAV-PHP.eB-CMV-EGFP or AAV2/8-CMV-EGFP (SR AAV-PHP.eB-CMV-EGFP and SR AAV2/8-CMV-EGFP, respectively; n = 3–4). Eyes were enucleated, fixed in 4% PFA at 1 month post-injection and cryosectioned. 1.3 × 10^5^ primary porcine RPE (pRPE) cells/well were transduced with 2.0 × 10^10^ vg of AAV-PHP.eB-CMV-EGFP (AAV-PHP.eB) or AAV2/8-CMV-EGFP (AAV2/8) and fixed in 4% PFA 48 h post-transduction (n = 4). (A, B, D, and E) EGFP fluorescence in retinas transduced with 3.0 × 10^7^ vg/eye AAV (note that D was exposed 20 times longer than A) was depicted in green, cones were labeled with ARR3 immunohistochemistry (magenta), and nuclei were counterstained with DAPI (blue; shown on the right side of the panels). (C and F) EGFP fluorescence in pRPE cells was depicted in green (note that F was exposed 5 times longer than C) and nuclei were counterstained with Hoechst (blue). (G and H) Mean EGFP fluorescence intensities in transduced retinas in the ONL (G) and in transduced pRPE cells (H) were quantified in cellSens. Arrowheads indicate EGFP^+^ RPE cells and arrows indicate cone-shaped cells (A and D). Arrows indicate EGFP and ARR3 double-labeled cells; that is, transduced cone photoreceptors (B and E). PS, photoreceptor segments. Scale bars represent 50 μm (D and F) and 10 μm (E). ∗∗∗∗p < 0.0001, ANOVA (G), t test (H).

A 5× dose curve of 6.0 × 10^6^, 3.0 × 10^7^, 1. 5 × 10^8^, and 7.5 × 10^8^ vg/eye of AAV-PHP.eB-CMV-EGFP and AAV2/8-CMV-EGFP was delivered subretinally (SR AAV-PHP.eB-CMV-EGFP and SR AAV2/8-CMV-EGFP, respectively) to murine eyes and EGFP expression analyzed at 1 month post-injection (n = 3–4). The AAV2/8 serotype was chosen as a control as this serotype is known to transduce both rod and cone photoreceptors and RPE cells highly efficiently.^10^^,^^20^^,^^45^^,^^46^ Robust EGFP expression in the photoreceptor layer and RPE and much lower EGFP expression in horizontal cells were observed in the transduced retinas (Figures 4A and 4D; note that labeled horizontal cells were not visible at the exposure level used in Figure 4). The inner retina (apart from horizontal cells) was not transduced (Figures 4A and 4D). EGFP fluorescence was present not only in rods but also in cone-shaped cells using both AAV serotypes (Figures 4A and 4D). We used ARR3 (cone marker) immunohistochemistry to label cones and confirmed colocalization of EGFP with ARR3 (Figures 4B and 4E). To minimize fluorescence signal interference from overlapping cells, colocalization of EGFP and ARR3 was performed in areas with lower transduction rates and therefore many fewer labeled cells (Figures 4B and 4E).

Notably, SR AAV-PHP.eB-CMV-EGFP resulted in much stronger EGFP expression levels compared to SR AAV2/8-CMV-EGFP. For example, Figure 4D was taken with a 20× exposure of Figure 4A, while using the same AAV dose of 3.0 × 10^7^ vg/eye. Figure S1 depicts EGFP fluorescence levels from retinas transduced with AAV doses of 3.0 × 10^7^ and 6.0 × 10^6^ vg/eye (n = 3). For SR AAV-PHP.eB-CMV-EGFP transduced retinas, a 3-ms exposure time was used (Figures S1A–S1C and S1G–S1L), while for SR AAV2/8-CMV-EGFP transduced retinas, equal (3 ms; Figures S1D–S1F and S1M–S1O), 20-fold (60 ms; Figures S1G–S1I), and 40-fold (120 ms; Figures S1P–S1R) exposure times were used. EGFP expression from SR AAV-PHP.eB-CMV-EGFP may have reached toxic levels in some parts of the retinas receiving 3.0 × 10^7^ - 7.5 × 10^8^ vg/eye doses of SR AAV-PHP.eB-CMV-EGFP as detected by the loss of photoreceptors and/or photoreceptor segments (Figure S2). Mean EGFP fluorescence intensities in the outer nuclear layer (ONL) of images from transduced retinas were quantified using cellSens. When transduced with 3.0 × 10^7^ vg/eye SR AAV-PHP.eB-CMV-EGFP or SR AAV2/8-CMV-EGFP, mean fluorescence intensity levels were 27,513.0 ± 4,232.3 and 566.4 ± 523.1, respectively (n = 3–4), which represents a 48.6-fold difference (p < 0.0001, ANOVA; Figure 4G). When transduced with 6.0 × 10^6^ vg/eye SR AAV-PHP.eB-CMV-EGFP or SR AAV2/8-CMV-EGFP, fluorescence intensity levels were 24,316.8 ± 4,111.2 and 155.6 ± 56.3, respectively, (n = 3–4), representing a 156.3-fold difference (p < 0.0001, ANOVA; Figure 4G).

### Transduction of primary porcine RPE cells

As the RPE was also EGFP^+^ in eyes transduced with both SR AAV-PHP.eB-CMV-EGFP and SR AAV2/8-CMV-EGFP (Figures 4A and 4D), we wanted to further explore and verify the transduction of RPE cells with AAV-PHP.eB. Primary porcine RPE (pRPE) cell culture is a convenient *in vitro* model for RPE cells (e.g., porcine eyes provide a sufficient amount of RPE cells; additionally, pRPE cells can easily be transduced with AAV).^47^^,^^48^ As such, we used this model; 1.3 × 10^5^ cells/well of pRPE cells were seeded in 8-well imaging chamber slides and transduced with 2.0 × 10^10^ vg/well of AAV-PHP.eB-CMV-EGFP or AAV2/8-CMV-EGFP (n = 4) 24 h later. pRPE cells were fixed and analyzed 48 h post-transduction, and EGFP expression analyzed and confirmed using EGFP fluorescence (Figures 4C and 4F). As AAV-PHP.eB-CMV-EGFP (Figures 4C and S3A–S3D) provided higher expression than the dose-matched AAV2/8-CMV-EGFP (Figures S3E–S3H); 5 times longer EGFP exposure times for AAV2/8-CMV-EGFP (Figures 4F and S3I–S3L) were used to compensate for the lower expression levels from this vector. Mean EGFP fluorescence intensity levels were quantified using cellSens; transduction with AAV-PHP.eB-CMV-EGFP and AAV2/8-CMV-EGFP resulted in 5,874.3 ± 668.5 and 1,192.6 ± 69.3 mean fluorescence intensities, respectively (n = 4, p < 0.0001, t test; Figure 3H); these represented a 4.93-fold difference.

### EGFP mRNA expression analysis

**Figure 5:**
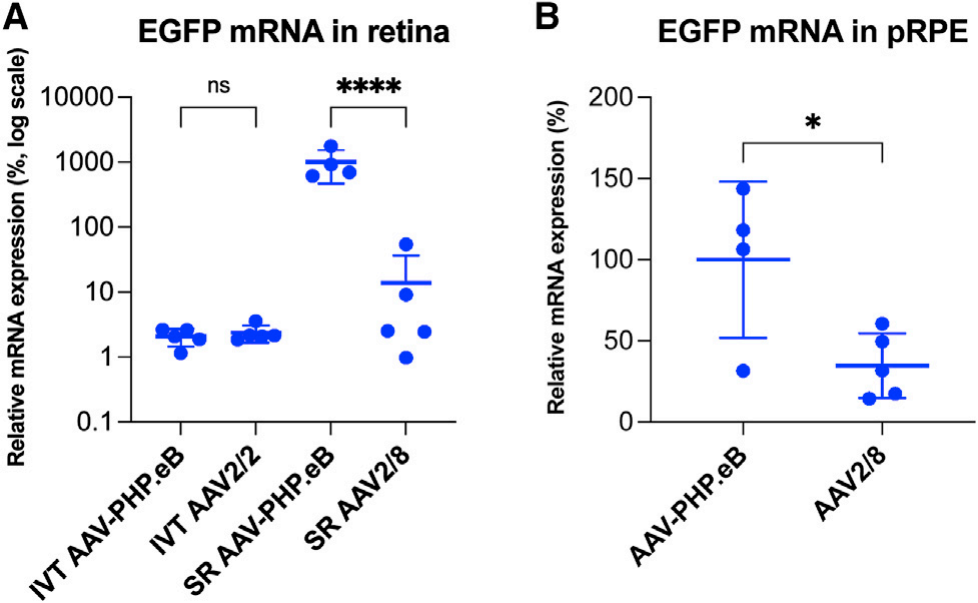
EGFP mRNA expression analysis in transduced retinas and primary porcine RPE cells Eyes were injected IVT with 7.5 × 10^8^ vg/eye of AAV-PHP.eB-CMV-EGFP (IVT AAV-PHP.eB) or AAV2/2-CMV-EGFP (IVT AAV2/2), or subretinally (SR) with 3.0 × 10^7^ vg/eye of AAV-PHP.eB-CMV-EGFP (SR AAV-PHP.eB) or AAV2/8-CMV-EGFP (SR AAV2/8; n = 5). Transduced retinas were harvested at 1 month post-injection. 1.00 × 10^5^ of pRPE cells/well were seeded in 48-well plates. After 24 h, pRPE cells were transduced with 1.00 × 10^9^ vg/well of AAV-PHP.eB-CMV-EGFP (AAV-PHP.eB; n = 4) or AAV2/8-CMV-EGFP (AAV2/8; n = 5) and cells harvested 24 h post-transduction. RNA was isolated and EGFP mRNA expression analyzed by qRT-PCR in transduced retinas (A) and pRPE cells (B). Relative expression of SR AAV-PHP.eB was assigned a value of 1,000; note the logarithmic scale on the y axis (A). Relative expression of AAV-PHP.eB in pRPE cells was assigned a value of 100 (B). ∗p < 0.05 (t test), ∗∗∗∗p < 0.0001, ANOVA.

As a second measure of EGFP expression levels, we determined EGFP mRNA expression levels in transduced retinas and pRPE cells. Quantitative reverse transcriptase PCR (qRT-PCR) was used to quantify EGFP mRNA expression levels in RNA isolated from IVT and SR transduced retinas and pRPE cells. For IVT delivery, 7.5 × 10^8^ vg/eye of AAV-PHP.eB-CMV-EGFP or AAV2/2-CMV-EGFP, and for SR delivery, 3.0 × 10^7^ vg/eye of AAV-PHP.eB-CMV-EGFP or AAV2/8-CMV-EGFP was used (n = 4–5). Transduced retinas were harvested at 1 month post-injection. In these experiments, SR AAV-PHP.eB-CMV-EGFP provided the highest expression level and was assigned a relative EGFP mRNA expression level of 1,000 (SD = ±529.2; Figure 5A). EGFP mRNA expression levels relative to SR AAV-PHP.eB-CMV-EGFP were 13.9 ± 22.9, 2.07 ± 0.6, and 2.36 ± 0.7 for SR AAV2/8-CMV-EGFP, IVT AAV-PHP.eB-CMV-EGFP, and IVT AAV2/2-CMV-EGFP, respectively (Figure 5A). Consequently, IVT AAV-PHP.eB-CMV-EGFP and IVT AAV2/2-CMV-EGFP provided similar EGFP mRNA expression levels (p = ns, ANOVA; Figure 5A). In contrast, SR AAV-PHP.eB-CMV-EGFP provided 71.9-fold higher expression compared to SR AAV2/8-CMV-EGFP (p < 0.0001, ANOVA; Figure 5A) in the analyzed retinas.

pRPE cells were transduced with 1.00 × 10^9^ vg/well (48-well plate) of AAV-PHP.eB-CMV-EGFP or AAV2/8-CMV-EGFP (n = 4–5) and harvested 24 h post-transduction. EGFP expression from AAV-PHP.eB-CMV-EGFP was assigned a relative EGFP mRNA expression level of 100 (SD = ±48.2; Figure 5B). Relative EGFP mRNA expression from AAV2/8-CMV-EGFP was 34.7 ± 20.1 (p < 0.05, t test; Figure 5B) (i.e., expression from AAV-PHP.eB-CMV-EGFP was 2.9-fold higher than from AAV2/8-CMV-EGFP) (Figure 5B).

### Glial cell activation

**Figure 6:**
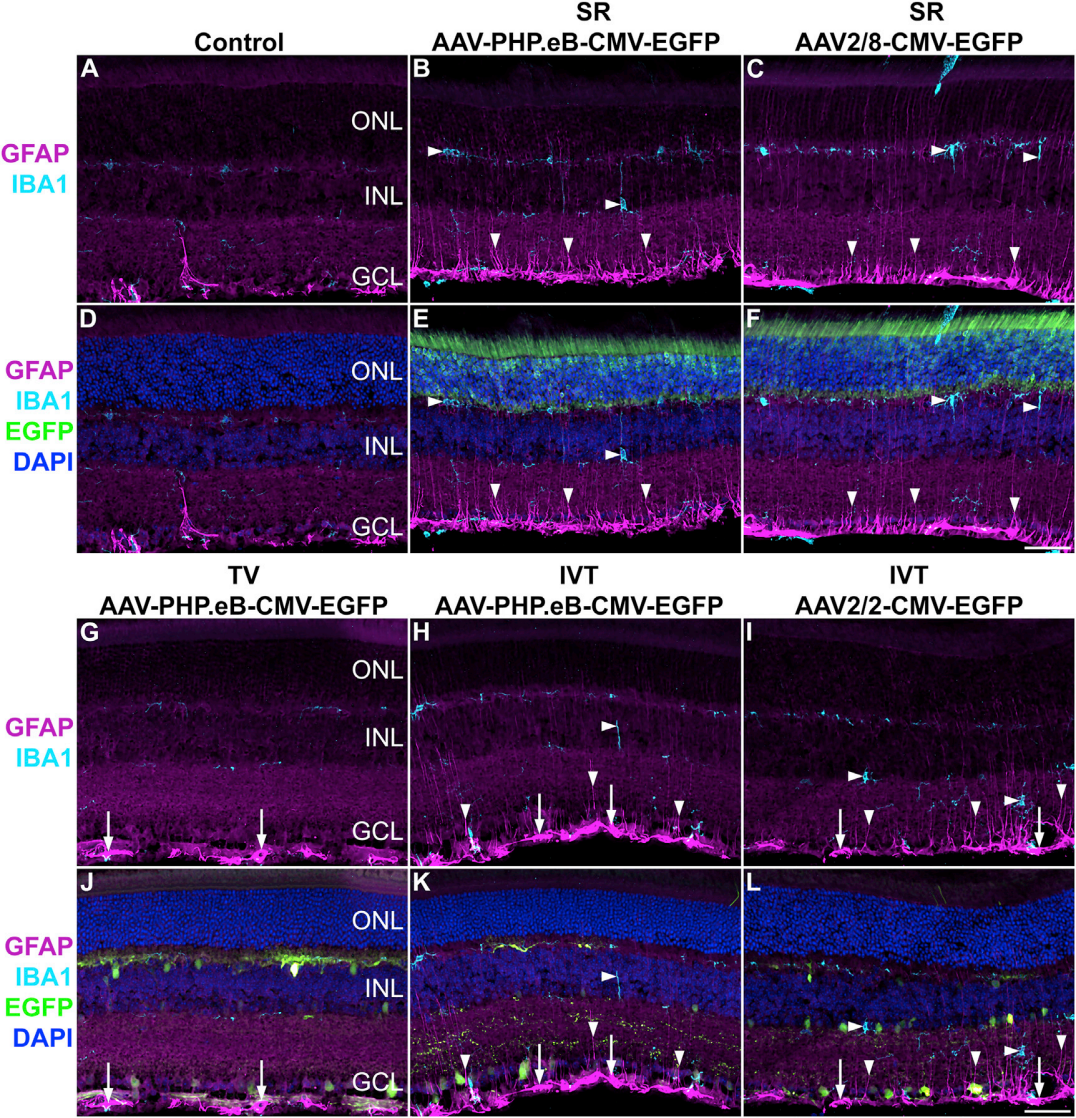
Glia cell activation Adult mice were injected SR with 3.0 × 10^7^ vg/eye of AAV-PHP.eB-CMV-EGFP or 3.0 × 10^7^ vg/eye of AAV2/8-CMV-EGFP (B, C, E, and F), or via the TV, with 5.0 × 10^10^ vg of AAV-PHP.eB-CMV-EGFP (G and J), or IVT with 7.5 × 10^8^ vg/eye of AAV-PHP.eB-CMV-EGFP (H andK) or 7.5 × 10^8^ vg/eye of AAV2/2-CMV-EGFP (I and L; n = 3–4); uninjected eyes were used as controls (A and D). Eyes were fixed in 4% PFA at 1-month post-injection and cryosectioned. EGFP fluorescence is depicted in green; sections were labeled with IBA1 (microglia marker, light blue) and GFAP (magenta) immunohistochemistry, and nuclei counterstained with DAPI (blue). GFAP and IBA1 labels (A–C and G–I), and GFAP, IBA1, EGFP, and DAPI labels (D–F and J–L) were overlaid. As SR AAV2/8-CMV-EGFP resulted in lower EGFP levels compared to SR AAV-PHP.eB-CMV-EGFP, and as the EGFP channel was used purely for the verification of transduction in these images, higher EGFP exposure times were used for SR AAV2/8-CMV-EGFP (F) versus SR AAV-PHP.eB-CMV-EGFP (E) images to clearly demonstrate transduction with SR AAV2/8-CMV-EGFP (F). Horizontal arrowheads indicate microglia cells and processes, downward arrowheads indicate Müller glia processes, and arrows indicate astrocytes. Scale bars (F and L) represent 50 μm.

**Figure 7:**
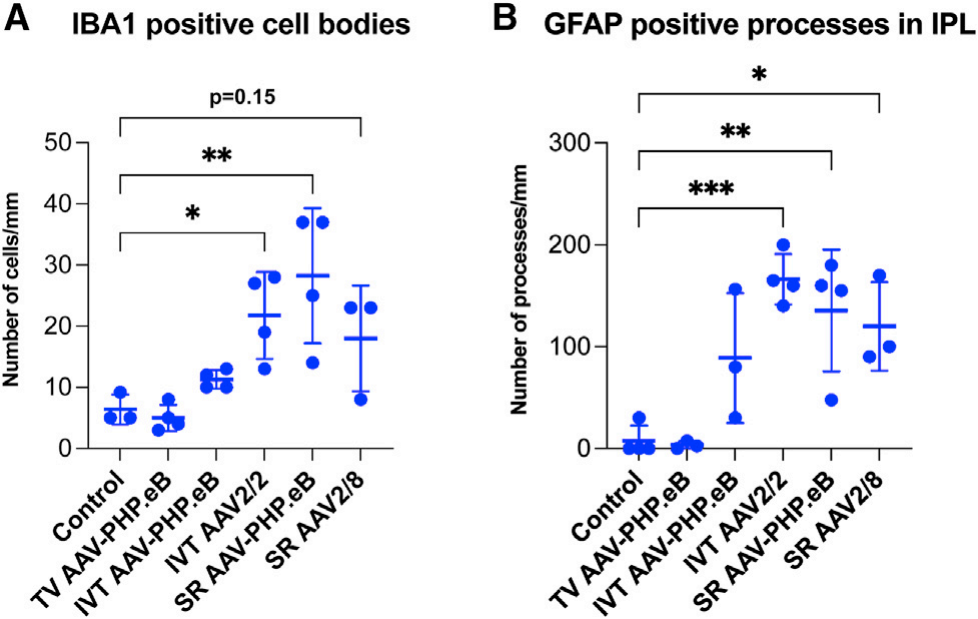
Quantification of glial cells and processes Adult mice were injected SR with 3.0 × 10^7^ vg/eye of AAV-PHP.eB-CMV-EGFP (SR AAV-PHP.eB) or 3.0 × 10^7^ vg/eye of AAV2/8-CMV-EGFP (SR AAV2/8), or via the TV with 5.0 × 10^10^ vg of AAV-PHP.eB-CMV-EGFP (TV AAV-PHP.eB), or IVT with 7.5 × 10^8^ vg/eye of AAV-PHP.eB-CMV-EGFP (IVT AAV-PHP.eB) or 7.5 × 10^8^ vg/eye of AAV2/2-CMV-EGFP (AAV2/2); uninjected eyes were used as controls. Eyes were fixed in 4% PFA at 1 month post-injection and cryosectioned. Sections were labeled with IBA1 and GFAP immunohistochemistries. IBA1^+^ cell bodies (A) and GFAP^+^ processes in the IPL were quantified using CellSens. ∗p < 0.05, ∗∗p < 0.01, ∗∗∗p < 0.001, ANOVA.

Glial cell activation associated with AAV administration has been observed at times in AAV delivery to the retina and depends on many factors, including the AAV serotype, AAV dose, and AAV quality and nature of the transgene.^49^^,^^50^ In a preliminary study, we assessed glial cell activation using ionized calcium binding adapter molecule 1 (IBA1) and glial fibrillary acidic protein (GFAP) immunohistochemistry in the AAV-transduced murine retinas. Retinas administered with 3.0 × 10^7^ vg/eye of SR AAV-PHP.eB-CMV-EGFP (Figures 6B and 6E), or SR AAV2/8-CMV-EGFP (Figures 6C and 6F), or 5.0 × 10^10^ vg of TV AAV-PHP.eB-CMV-EGFP (Figures 6G and 6J), or 7.5 × 10^8^ vg/eye of IVT AAV-PHP.eB-CMV-EGFP (Figures 6H and 6K) or AAV2/2-CMV-EGFP (Figures 6I and 6L) were analyzed in these experiments (n = 3–4). Uninjected eyes were used as controls (n = 3; Figures 6A and 6D). Retinas were analyzed at 1 month post-AAV delivery. GFAP^+^ astrocytes and resting IBA1^+^ microglia characterized the uninjected control eyes (Figures 6A and 6D). Both SR (Figures 6B, 6C, 6E, and 6F) and IVT (Figures 6H, 6I, 6K, and 6L) delivery of AAV-PHP.eB, AAV2/2 and AAV2/8 serotypes resulted in moderate activation of glia cells: (1) Microglia cells exhibited increased IBA1 immunostaining intensity, increased branching, and longer processes. The number of IBA1^+^ cells increased significantly for IVT AAV2/2-CMV-EGFP (n = 4, p < 0.05, ANOVA), SR AAV-PHP.eB-CMV-EGFP (n = 4, p < 0.01, ANOVA), and SR AAV2/8-CMV-EGFP (n = 3, p = 0.15, ANOVA; Figure 7A) compared to the uninjected control eyes (n = 3). (2) Müller cell processes displayed increasing amounts of GFAP expression from the ONL to the GCL, with the strongest labeling intensity observed in the inner retina. The number of GFAP-labeled processes in the inner plexiform layer (IPL) increased significantly for IVT AAV2/2-CMV-EGFP (n = 4, p < 0.001, ANOVA), SR AAV-PHP.eB-CMV-EGFP (n = 4, p < 0.01, ANOVA), and SR AAV2/8-CMV-EGFP deliveries (n = 3, p < 0.05, ANOVA; Figure 7B) compared to the uninjected control eyes (n = 4). (3) GFAP staining intensity was increased in astrocytes (Figures 5B, 5C, 5E, 5F, 5H, 5I, 5K, and 5L). Overall, similar glial activation was observed between AAV-PHP.eB and the benchmark serotypes, except for TV delivery. Notably, there was minimal glia activation with TV AAV-PHP.eB-CMV-EGFP; only GFAP intensity in astrocytes increased (Figure 6D). However, it is possible that glial activation would intensify if the dose of TV AAV-PHP.eB-CMV-EGFP was increased.

## Discussion

In this study, the transduction profile of AAV-PHP.eB was investigated in the mouse retina using a CMV-EGFP expression cassette.^23^ *In vivo* directed evolution of the AAV9 capsid by Cre-REcombinase-based AAV Targeted Evolution (CREATE) was used to develop AAV-PHP.B^51^ and AAV-PHP.eB,^23^ which efficiently targets various CNS neurons and astrocytes in the mouse. The increased ability to cross the blood-brain barrier, when delivered via systemic administration, is at least one of the reasons underlying the increased efficacy of gene delivery with these serotypes; the glycosylphosphatidylinositol-anchored protein called lymphocyte antigen 6 complex, locus A (LY6A) has been identified as an essential receptor for the modified capsids of AAV-PHP.B and AAV-PHP.eB.^36^^,^^52^^,^^53^ Indeed, crossing of the blood-brain barrier is dependent on the species-specific expression of LY6A in CNS endothelia, which is present only in certain mouse strains^53^—for example, in C57BL/6J, used in the study by Simpson et al.^35^ and 129 strains, used in the present study—but not in, for example, B6C3 or BALB/cJ.^36^^,^^52^^,^^54^ In NHPs, in which there is no direct homolog of LY6A, limited transduction was achieved in the CNS following intravascular administration of AAV-PHP.B,^55^^,^^56^ but broad cortical and spinal cord transduction was attained following intrathecal administration.^56^

The parental serotype of AAV-PHP.B (i.e., AAV9) has been shown to transduce the retina in neonatal mice when administered via systemic delivery.^28^^,^^30^^,^^31^^,^^33^^,^^34^ Simpson et al.^35^ established aspects of the transduction profile of AAV-PHP.eB in the C57BL/6J murine retina, although, in contrast to the present study, minimal transgene expression was found after IVT delivery. Furthermore, in the Simpson et al. study,^35^ the comparative efficacy of AAV-PHP.eB to benchmark AAV serotypes, which transduce the mammalian retina efficiently, was not explored, nor was the identification of transduced retinal cell types using specific cell markers. In the present study, AAV-PHP.eB-CMV-EGFP was delivered via systemic TV, IVT, and SR injections in adult mice. Control AAV serotypes for IVT and SR delivery included AAV2/2^18^^,^^37^, ^38^, ^39^, ^40^, ^41^ and AAV2/8,^10^^,^^20^^,^^45^^,^^57^ respectively. In addition, EGFP signal was colocalized with retinal cell-type-specific markers using immunohistochemistry.

Our results indicate that AAV-PHP.eB transduces RGCs efficiently at the doses used when administered both via TV and IVT (TV: 5.0 × 10^10^ vg/mouse and IVT: 7.5 × 10^8^ vg/eye); the number of EGFP^+^ cells in the GCL (and INL) were similar (Figures 1 and 2). However, following IVT delivery, EGFP fluorescence intensities varied significantly in different areas of the retinas (up to a ∼115-fold difference in fluorescence intensity; Figure 3), although the optic nerve head area consistently expressed the transgene at high levels. IVT delivery often produces uneven transduction of the retina.^35^^,^^58^ With TV delivery, a more even distribution and pan-retinal coverage of EGFP^+^ cells was observed in the retina, although there was a tendency for higher EGFP fluorescence intensities (+39.3%) toward the periphery (Figure 3). Apart from RGC cell bodies, RGC dendrites and axons were also positive for EGFP after TV and IVT AAV-PHP.eB-CMV-EGFP administration (Figures 1 and 3). Indeed, visualization of EGFP^+^ RGC axons was possible from the retina, via the optic nerve and optic tract to thalamic nuclei and the colliculus superior post-IVT administration of AAV-PHP.eB-CMV-EGFP (Figure 3). AAV-PHP.eB also transduced some amacrine (PAX6^+^), bipolar (VSX2^+^) and horizontal cells (and their processes) in the INL when delivered via SR or IVT, as evaluated by EGFP fluorescence; in contrast, Müller cells (CRALBP^+^) were not transduced (Figure 2). Notably, TV and IVT routes of delivery transduced the inner retina negligibly, with minimal photoreceptor labeling, which was observed mostly in very highly transduced areas. Overall, the cell-specific tropisms of TV and IVT administration of AAV-PHP.eB-CMV-EGFP were similar (Figures 1 and 2) to each other and to that of IVT AAV2/2-CMV-EGFP, a serotype commonly used for IVT delivery to RGCs.^18^^,^^37^, ^38^, ^39^^,^^58^^,^^59^

In contrast to the broad transduction of RGCs via both IVT and TV delivery in our study (Figures 1, 2, and 3), minimal transduction of RGCs was found in the Simpson et al. study.^35^ Indeed, Simpson and colleagues^35^ found limited transduction of retinal cells, other than horizontal cells (which were transduced efficiently), when AAV-PHP.eB was delivered systemically and minimal transduction of any cells when delivered via IVT. It is possible that the effective AAV dose or achieved expression level of CBA-tdTomato (the fluorescent protein used in their study) was not sufficient to provide detectable levels of tdTomato in RGC and INL cells.^35^ However, supporting our results, significant GCL (and INL) transduction has been demonstrated following systemic delivery of AAV-PHP.B (the parental serotype of AAV-PHP.eB).^46^ In our study, TV AAV-PHP.eB-CMV-EGFP was very efficient at transducing horizontal cells, which agrees with the study by Simpson and colleagues.^35^ In addition, we quantified EGFP mRNA expression levels with qRT-PCR and found them to be similar between IVT AAV-PHP.eB-CMV-EGFP and IVT AAV2/2-CMV-EGFP transduced retinas (Figure 5A); such quantification has not been performed previously. The quantification of EGFP at the mRNA (Figure 5A) and protein levels (Figure 1) correspond with each other and suggest similar overall efficacies between AAV-PHP.eB and AAV2/2 serotypes when delivered via IVT.

Of note, in agreement with Simpson and colleagues,^35^ SR AAV-PHP.eB-CMV-EGFP resulted in very high levels of EGFP expression in photoreceptor and RPE cells in our study (Figure 4). The quantification of both histology and mRNA expression levels in SR transduced retinas suggests significantly higher levels of EGFP expression provided by AAV-PHP.eB compared to AAV2/8 (48.6-fold higher EGFP fluorescence intensity by histology and 71.9-fold greater retinal mRNA levels at 3.0 × 10^7^ vg/eye dose; Figures 4 and 5A). Note that the mRNA was obtained from retinas (without RPE), while the fluorescence was measured in the ONL; as such, the data reflect expression levels in photoreceptor cells. The increase in expression levels in the ONL between AAV-PHP.eB and AAV2/8 was similar in magnitude to that found in the brain with AAV-PHP.B^51^ and AAV-PHP.eB^23^ compared to AAV9. While the enhanced transduction is explained by the permissive blood-brain barrier crossing in the brain, it is unclear why AAV-PHP.eB transduces photoreceptors (and RPE) so efficiently via direct (i.e., SR) delivery. Using ARR3 immunocytochemistry, we demonstrated transduction of cone photoreceptors, in addition to rods, following SR administration of AAV-PHP.eB. Using pRPE primary cell culture, we determined that RPE cells were readily transduced by AAV-PHP.eB-CMV-EGFP *in vitro*, indicating that these cells are targeted by AAV-PHP.eB. As such, the EGFP protein found *in vivo* in the RPE cells in SR AAV-PHP.eB-CMV-EGFP and SR AAV2/8-CMV-EGFP transduced mouse retinas was likely due to *in situ* expression of EGFP—that is, bona fide transduction of RPE cells rather than that due to phagocytosis of transduced photoreceptors. Indeed, AAV2/8 is known to transduce the RPE,^57^ and we suggest that the same is the case for AAV-PHP.eB. EGFP protein (determined from fluorescence intensity) and EGFP mRNA expression levels in transduced pRPE cell cultures were 4.9-fold (Figure 4B) and 2.9-fold (Figure 5B) higher, respectively, when transduced with AAV-PHP.eB-CMV-EGFP compared to dose-matched AAV2/8-CMV-EGFP. While these differences were lower compared to the data obtained in transduced retinas, the trends were similar; that is, higher EGFP expression levels were achieved in RPE cells both *in vitro* and *in vivo* when transduced with AAV-PHP.eB compared to dose-matched AAV2/8.

As noted above, the main feature of the AAV-PHP.B serotype family is efficient systemic delivery to the CNS.^53^^,^^55^ As this is mouse specific, systemic delivery of AAV-PHP.B is inefficient in NHPs.^55^^,^^56^ However, the significant CNS transduction following intrathecal administration in macaques^56^ suggests that, similar to mice, intraocular SR and IVT deliveries of AAV-PHP.eB may transduce the retina efficiently in NHPs and humans. Indeed, retinal organoids developed from human induced pluripotent stem cells (iPSCs) were also transduced efficiently with AAV-PHP.eB.^60^ As such, further studies are needed to determine the efficacy and transduction profile of IVT and SR AAV-PHP.eB deliveries in NHP retinas.

In a preliminary study, we also analyzed the impact of AAV-PHP.eB-CMV-EGFP transduction on glia cell activation in the retina. We found moderate activation of astrocytes, Müller cells, and microglia following SR and IVT deliveries of AAV-PHP.eB-CMV-EGFP. Glia activation from IVT and SR AAV-PHP.eB-CMV-EGFP was similar to the benchmark AAV serotypes (i.e., SR AAV2/8-CMV-EGFP and IVT AAV2/2-CMV-EGFP). In general, glia cell activation is a multi-factorial event determined by various factors such as AAV delivery route, AAV serotype, AAV purity, and transgene and promoter usage.^49^^,^^50^ EGFP is known for its toxicity in the retina,^20^ and the CMV promoter provides high levels of EGFP expression. Our results suggest that AAV-PHP.eB may be tolerated to the same extent as AAV2/2 and AAV2/8 in the retina. Interestingly, TV AAV-PHP.eB-CMV-EGFP had a very low glia activation profile in our experiments (Figures 6 and 7). It appears that TV delivery may have some properties that could result in less stress to the retina in TV versus SR or IVT deliveries of AAV and may have accounted for the lower glial activation found in the present study. These include (1) no physical injury to the retina with TV versus SR or IVT deliveries; (2) AAV particles may take longer time to cross the blood-brain barrier (TV) compared to the direct retinal injections (SR, IVT), which may result in attenuated and lower local AAV levels in the retina; and (3) AAV impurities are unlikely to cross the blood-brain barrier or may be diluted out. Indeed, while matching the AAV dose between TV versus intraocular delivery is not possible, increasing the TV dose may increase glial activation, which could at some point equal the levels found at IVT and SR deliveries.

### Summary

Our study indicates that AAV-PHP.eB is a versatile and efficient AAV serotype for retinal transduction, targeting both the inner and outer retina depending on the delivery route in mice. AAV-PHP.eB targets cell types involved in many common retinal degenerations such as RGCs, photoreceptors, and RPE with high efficiency. In particular, our study suggests that systemic delivery of AAV-PHP.eB provides even and pan-retinal transduction of RGCs and horizontal cells. However, the approximately 70-fold higher dose used for TV versus IVT delivery and the extensive transduction of other organs with TV AAV-PHP.eB^23^^,^^54^ suggests experimental rather than translational utility for systemic AAV-PHP.eB in the context of targeting the retina. In addition, SR delivery of AAV-PHP.eB-CMV-EGFP resulted in 50- to 70-fold higher expression levels compared to AAV2/8-CMV-EGFP, which is typically considered a benchmark serotype for targeting photoreceptors.^20^^,^^61^^,^^62^ Such efficacy may enable a significant reduction in AAV dose, while still achieving equivalent levels of transgene expression. The evolution of AAV technology continues and is hallmarked by the frequent emergence of new and promising AAV serotypes. For example, systemic delivery of a gene-replacement therapy with AAV-PHP.B (the parental serotype of AAV-PHP.eB) provided significant benefit in an Ndufs4 knockout mouse model of Leigh syndrome,^59^ where previous attempts using AAV1^63^ and AAV2/9^64^ serotypes resulted in less improvement. As such, the utility of AAV-PHP.B may represent a significant step toward the clinic for Leigh syndrome. Another novel AAV serotype, that is, AAV44.9, has been reported to be exceptionally efficient at transducing cone and rod photoreceptors in both mice and macaques.^61^ Our study highlights the efficacy of AAV-PHP.eB at transducing RGCs, photoreceptors, and RPE cells in mice. To fully explore the potential utility of AAV-PHP.eB for retinal gene delivery, it would be pivotal to determine the efficacy of this serotype with intraocular delivery in NHP eyes. Ultimately, some of the above (AAV-PHP.B, AAV-PHP.eB, and AAV44.9) and future AAV serotypes may have the potential to challenge and even replace the current benchmark set of AAV serotypes in both preclinical and clinical settings.

## Materials and methods

### Constructs and AAV production

In this study, the previously described pAAV-CMV-EGFP plasmid was used^65^ to generate AAV-PHP.eB-CMV-EGFP, AAV2/2-CMV-EGFP, and AAV2/8-CMV-EGFP recombinant virus vectors. AAV-PHP.eB, AAV2/2, and AAV2/8 capsid plasmids were obtained from Addgene (plasmid #103005),^23^ Agilent Technologies Ireland (Cork, Ireland), and Prof. James M. Wilson (Perelman School of Medicine, University of Pennsylvania; Addgene plasmid #112864), respectively. Recombinant AAVs were generated using a triple transfection method,^66^ and then purified by differential precipitation and cesium gradient centrifugation.^65^^,^^67^^,^^68^ Genomic titres (viral genomes/mL; vg/mL) were determined by qPCR using EGFP-specific primers (forward: 5′-TTCAAGGAGGACGGCAACATCC, reverse: 5′-AGCTGCACGCTGCCGTCCTC)^69^ and were between 5.0 × 10^11^–5.0 × 10^13^, depending on serotype.

### Animals and AAV delivery

Adult (20- to 22-week-old) wild-type 129 S2/SvHsd mice (Harlan UK, Oxfordshire, UK) were used in this study; the 129 strains are permissive to PHP.B transduction across the blood-brain barrier,^36^ which was confirmed in our 129 line in a preliminary study. Animals were maintained under specific pathogen free (SPF) housing conditions and both sexes were used for experiments. Animal welfare complied with the Directive 2010/63/EU; Protection of Animals Used for Scientific Purposes, Regulations 2012 [S.I. no. 543 of 2,012] and the Association for Research in Vision and Ophthalmology (ARVO) Statement for the Use of Animals in Ophthalmic and Vision Research. The animal studies were approved by the Animal Research Ethics Committee, Trinity College Dublin. Various doses of AAV were delivered via subretinal and intravitreal injections in 3.0 μL PBS (supplemented with 0.001% Pluronic F68)/eye as previously described.^18^^,^^65^ Systemic delivery was performed via TV injection of 250 μL AAV in PBS supplemented with 0.001% Pluronic F68.

### Primary porcine RPE cell culture

pRPE cells were obtained from fresh pig eyes^47^ purchased from an abattoir. pRPE cells were grown in DMEM medium supplemented with 20% and 50 μg/mL gentamicin.

1.00 × 10^5^ and 1.30 × 10^5^ pRPE cells/well were plated in 48-well plates (mRNA analysis) and in 8-well imaging chamber slides (Miltenyi Biotec; histology analysis), respectively. After 24 h, cells were transduced with 1.00 × 10^9^ vg/well AAV in DMEM supplemented with 2% fetal bovine serum (FBS). FBS levels were increased to 20% 4 h post-transduction. Cells in 48-well plates were harvested 24 h post-transduction for RNA purification, while cells in chamber slides were fixed 48 h post-transduction.

### EGFP mRNA expression analysis

RNA was purified using the RNeasy mini kit (Qiagen, Manchester, UK) and EGFP mRNA expression quantified with qRT-PCR (QuantiTect SYBR Green RT-PCR Kit, Qiagen) using EGFP-specific primers (forward: 5′-TTCAAGGAGGACGGCAACATCC, reverse: 5′-AGCTGCACGCTGCCGTCCTC) and Actb as internal control.

### Histology, microscopy, and quantification

Mice were sacrificed, eyes enucleated, and brains removed and fixed in 4% paraformaldehyde in PBS at 4°C overnight; then, samples were washed in PBS (3 × 10 min). Retinal wholemounts were immediately stained after the wash steps. For cryosectioning, samples were cryoprotected in 10%, 20%, and 30% sucrose in PBS, embedded in OCT (VWR International, Dublin, Ireland), cryosectioned (12 μm), thaw mounted onto polysine slides (Thermo Fisher Scientific, Waltham, MA), and stored at −20°C. Retinal sections adjacent to the optic nerve head (±200 μm), optic nerves within 5 mm of the optic nerve head, and brain sections at Bregma −0.3 mm, Bregma −2.7 mm and Bregma −3.2 mm were used in this study. pRPE cells were fixed in 4% paraformaldehyde at room temperature for 20 min and then washed in PBS (3 × 10 min).

For immunohistochemistry and immunocytochemistry, slides were blocked in 5% donkey serum, 0.3% Triton X-100 in PBS (blocking solution) for 2 h at room temperature, and then incubated with primary antibodies (Table 1) in blocking solution at 4°C overnight and with corresponding secondary antibodies conjugated with Cy3 and AF647 (Jackson ImmunoResearch Europe, Ely, UK) in 1:400 dilution in blocking solution at room temperature for 2 h. Washes after the primary and secondary antibody incubation steps were carried out in PBS (3 × 10 min). Finally, nuclei were counterstained with DAPI for tissues and Hoechst for pRPE cells and samples washed in PBS (2 × 10 min).

**Table 1:** Antibodies used

Ab target	Ab species	Dilution used	Source	Catalog no.
ARR3	rabbit	1:200	Merck	AB15282
VSX2 (CHX10)	mouse	1:200	Santa Cruz	SC-374151
CRALBP	mouse	1:200	Abcam	AB15051
EGFP	chicken	1:1,000	Abcam	Ab13970
GFAP	chicken	1:500	Abcam	Ab4674
IBA1	rabbit	1:200	Wako	019-19741
NeuN	rabbit	1:200	Abcam	AB177487
PAX6	rabbit	1:200	Biolegend	PRB-278P
RBPMS	guinea pig	1:400	Millipore	ABN1376

Fluorescent microscopy was carried out using an Olympus IX83 (Mason Technology, Dublin, Ireland) inverted motorized microscope (cellSens Dimension version 1.9 software) equipped with a SpectraX LED light source (Lumencor, Mason Technology) and an Orca-Flash4.0 LT PLUS/sCMOS camera (Hamamatsu, Mason Technology). Multi-channel grayscale images were assigned with fluorescence colors and channels superimposed. Pan-retinal images were produced from images with lateral frames stitched together in cellSens. The quantification of cell numbers and fluorescence intensities were carried out in cellSens. EGFP-labeled cells, IBA1-labeled cells, and GFAP-labeled cell processes were counted manually in a 300-μm (100 μm for processes) wide frame; 2 areas/section and 4 sections/eye (sections at least 100 μm apart) were quantified for each sample. Mean fluorescence intensity (on a 16-bit scale) was measured in selected areas; 1 area/section and 4 sections/eye (sections at least 100 μm apart) were used for each eye, 10–16 areas/region of interest were selected in wholemounts, and 2 areas/well were analyzed for pRPE cells. Representative images for figures were exported from cellSens as individual fluorescence channels and post-processed in Photoshop (Adobe Systems Software Ireland, Dublin). In a given observation method, the same settings/operations were applied to all of the images both in cellSens and Photoshop, except for EGFP levels. EGFP levels varied significantly in the samples; therefore, a range of EGFP exposure levels were used. These are noted in the results section and/or figure legends.

### Statistical analysis

One-way ANOVA (with Dunnett’s multiple comparisons test) and unpaired t test were carried out using Prism version 9.3.1 (GraphPad; San Diego, CA, USA); p < 0.05 was considered statistically significant.

## Supplemental information

Document S1. Figures S1–S3

Document S2. Article plus supplemental information
