# Supplementary material for: AAV-PHP.eB transduces both the inner and outer retina with high efficacy in mice
Source: Mol Ther Methods Clin Dev. 2022 Mar 28;25:236–49. doi: 10.1016/j.omtm.2022.03.016 (PMC9018541; doi:10.1016/j.omtm.2022.03.016)
Supplement: Document S1. Figures S1–S3 [file mmc1.pdf]

OMTM, Volume *25*

Supplemental information

AAV-PHP.eB transduces both the inner and outer retina with high efficacy in mice

Arpad Palfi, Naomi Chadderton, Sophia Millington-Ward, Iris Post, Pete Humphries, Paul F. Kenna, and G. Jane Farrar

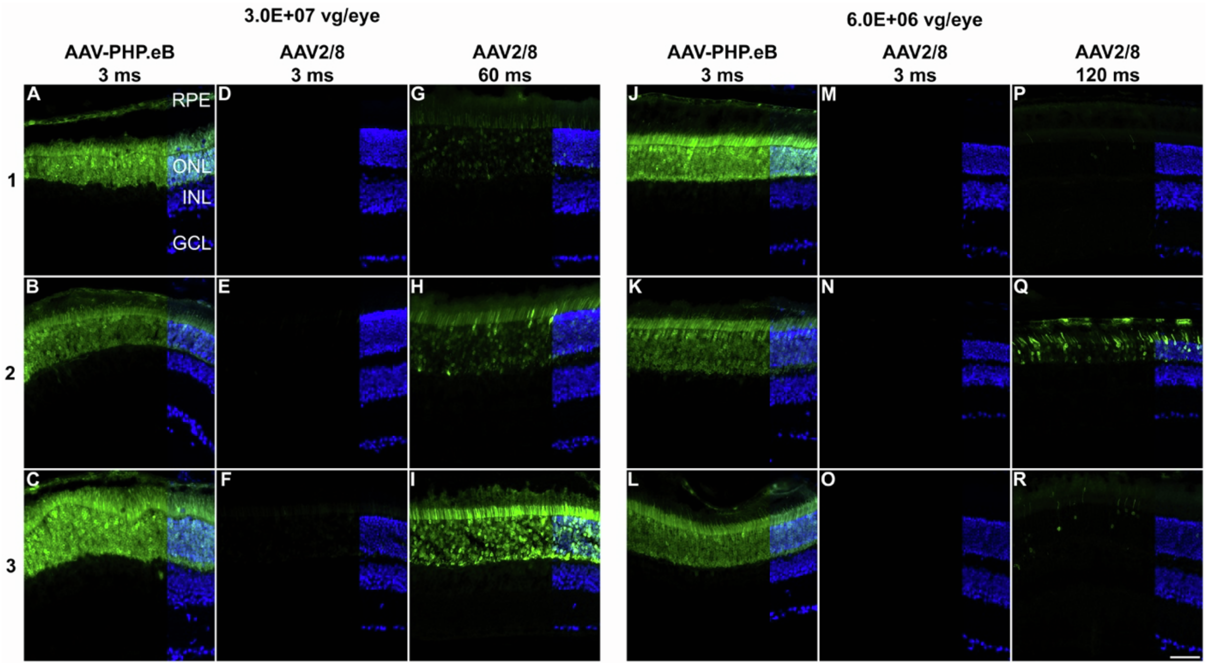

Fig. S1. Comparison of EGFP fluorescence intensities in retinas injected subretinally with AAV-PHP.eB-CMV-EGFP and AAV2/8-CMV-EGFP

Adult mouse eyes were injected subretinally with 3.0E+07 (A-I) and 6.0E+06 (J-R) vg/eye of AAV-PHP.eB-CMV-EGFP or AAV2/8-CMV-EGFP (n=3). Eyes were enucleated, fixed in 4% pfa at 1-month post-injection and cryosectioned. Expression of EGFP (green) was captured using 3 ms (A-F and J-O), 60 ms (G-I) and 120 ms (P-R) exposure times. Nuclei were counterstained with DAPI (blue; shown on the right side of the panels). RPE: retinal pigment epithelium, ONL: outer nuclear layer, INL: inner nuclear layer, GCL: ganglion cell layer. Scale bar (R) represent 50 µm.

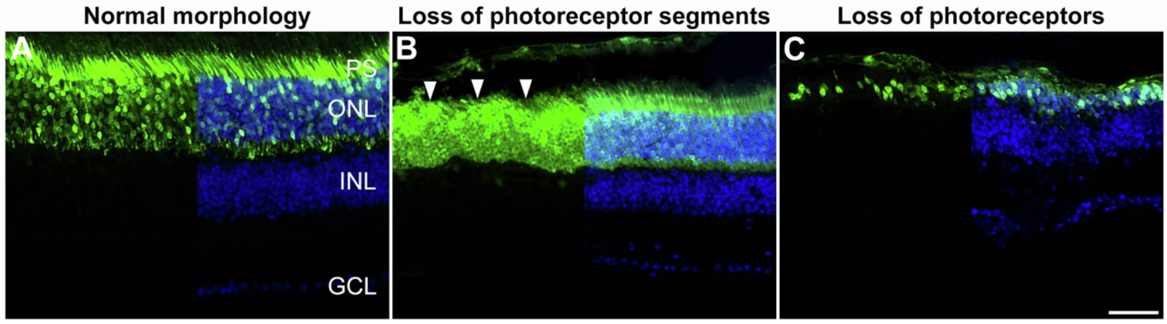

Fig. S2. Potential ONL damage caused by EGFP overexpression

Adult mouse eyes were injected subretinally with 3.0E+07 vp/eye of AAV-PHP.eB-CMV-EGFP; n=3. Eyes were enucleated, fixed in 4% pfa at 1-month post-injection and cryosectioned. A: area with normal morphology; B: area with loss of photoreceptor segments; C: area with loss of photoreceptors. EGFP fluorescence in retinas was depicted in green and nuclei counterstained with DAPI (blue; shown on the right half of the panels). Arrowheads point to a region with marked loss of photoreceptor segments (PS). ONL: outer nuclear layer, INL: inner nuclear layer, GCL: ganglion cell layer. Scale bar (C) represents 50 µm.

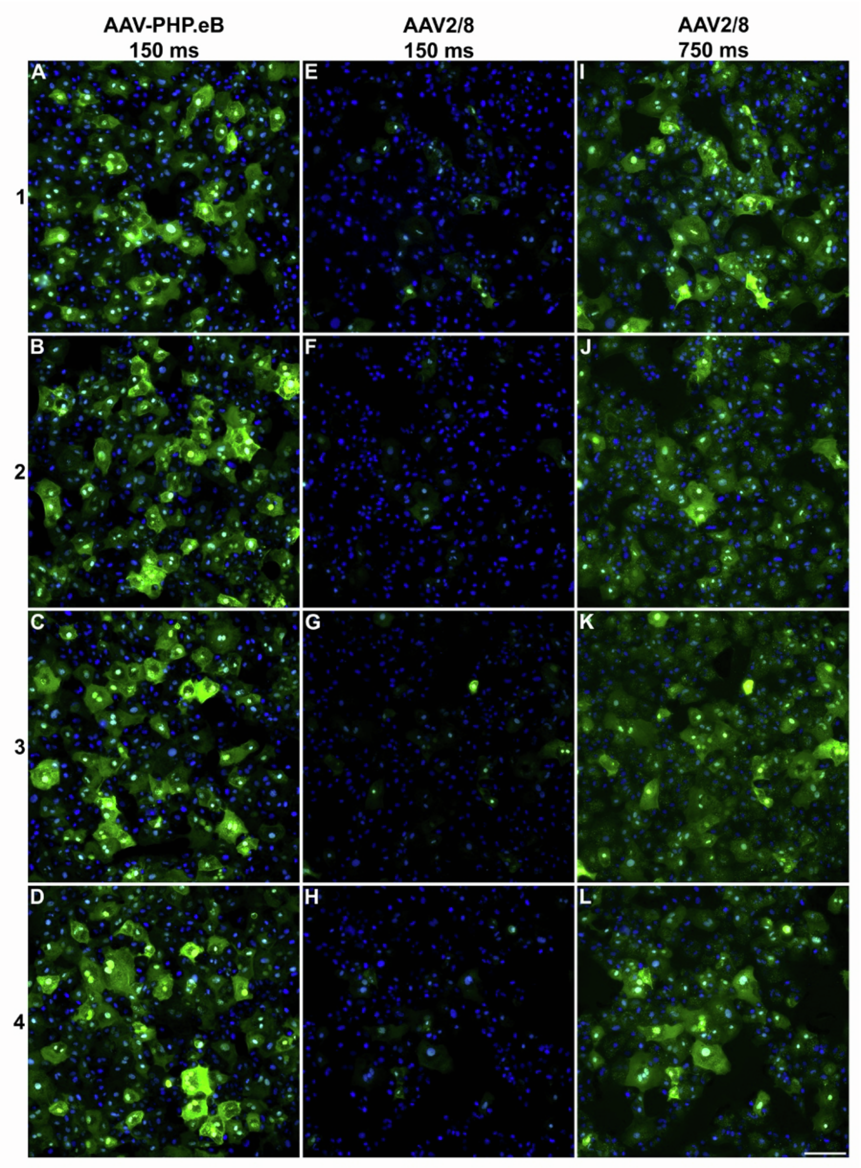

Fig. S3. Comparison of EGFP fluorescence levels in primary porcine RPE cells transduced with AAV-PHP.eB-CMV-EGFP and AAV2/8-CMV-EGFP

1.3E+05 primary porcine RPE (pRPE) cells/well were transduced with 2.0E+10 vg of AAV-PHP.eB-CMV-EGFP or AAV2/8-CMV-EGFP and fixed in 4% pfa 48 h post-transduction (n=4). EGFP fluorescence was depicted in green using 150 ms exposure for AAV-PHP.eB-CMV-EGFP (AAV-PHP.eB; A-D), and 150 ms (E-H) or 750 ms (I-L) exposures for AAV2/8-CMV-EGFP (AAV2/8); nuclei were counterstained with Hoechst. Scale bar (L): 100 µm. Note, that image C is also shown in Fig. 4 C, while image F (low exposure version of J) and J are also shown on Fig. 4 F.
